# Deep learning recommendation algorithm based on semantic mining

**DOI:** 10.1371/journal.pone.0274940

**Published:** 2022-09-26

**Authors:** Yongxin Huang, Hezheng Wang, Rui Wang

**Affiliations:** 1 The China Academy of Information and Communications Technology (CAICT), Beijing, China; 2 China Information and Communication Technology, Beijing, China; 3 University of Chinese Academy of Sciences, Beijing, China; Menoufia University, EGYPT

## Abstract

This paper proposes Deep Semantic Mining based Recommendation (DSMR), which can extract user features and item attribute features more accurately by deeply mining the semantic information of review text and item description documents recommend. First, the proposed model uses the BERT pre-training model to process review texts and item description documents, and deeply mine user characteristics and item attributes, which effectively alleviates the problems of data sparseness and item cold start; Then, the forward LSTM is used to pay attention to the changes of user preferences over time, and a more accurate recommendation is obtained; finally, in the model training stage, the experimental data are randomly divided into 1 to 5 points, 1:1:1:1:1. Extraction ensures that the amount of data for each score is equal, so that the results are more accurate and the model is more robust. Experiments are carried out on four commonly used Amazon public data sets, and the results show that with the root mean square error as the evaluation index, the error of DSMR recommendation results is at least 11.95% lower on average than the two classic recommendation models based only on rating data. At the same time, it is better than the three latest recommendation models based on review text, and it is 5.1% lower than the best model on average.

## 1 Introduction

The recommendation system has received great attention since its birth, and researchers have proposed many excellent algorithms to improve the efficiency and accuracy of recommendation. Deep learning is the application of deep learning models on the basis of traditional recommendation algorithms to mine deep-level user preference features, which further improves the accuracy of recommendation. Early algorithms mainly used rating data for recommendation. With the sharp increase in the number of users and items, problems such as data sparsity and cold start became more and more prominent, which became the main reason to limit the further improvement of recommendation accuracy. E-commerce not only brings a lot of commodity information, but also generates a lot of comment information. Comments include information on whether users are satisfied with the functions and quality of commodities [1]. Making full use of review information can accurately obtain user preferences and comprehensive product attributes, effectively alleviate data sparsity and cold start problems, and make recommendations more accurate.

Initially, researchers tried to use review texts for topic modeling [2–9], achieving higher prediction accuracy than models using only rating data. However, this method only focuses on the topic index, ignoring the semantic content, and usually expresses the comments as a bag of words, ignoring the context information [10], thus limiting the further improvement of the prediction accuracy. In recent years, many studies have begun to combine deep learning with review texts, proposing many excellent algorithms, and obtaining recommendation results with higher accuracy than methods based on topic modeling. References [11–14] concatenate multiple reviews into a long document and use convolutional neural networks to learn useful features from review texts. However, document-based modeling connects all comments to the same document indiscriminately, without distinguishing the different importance of different comments, which is not conducive to extracting effective features [15]. Therefore, researchers began to use the review-based modeling method, that is, model each review individually, and finally aggregate the features of each review into a total feature. The literatures [15–17] are all based on review modeling, and all use the attention mechanism to distinguish the importance of different reviews, and obtain a higher recommendation accuracy than the model based on document modeling accuracy.

To sum up, we have noticed the limitations of many current works: 1) Many models still use CNN to extract user and item features in reviews, which can only capture local features, and cannot effectively extract features from long sequences of text. It limits the improvement of recommendation accuracy. 2) In review-based models, many works do not consider that users’ interests and preferences will change over time [11–16], but treat past preferences and recent preferences equally. 3) The above-mentioned excellent models that use comment text to improve recommendation accuracy do not use comment text and also pay attention to the use of item description documents. The item description documents contain a more comprehensive introduction to the attributes of the items. Item cold start plays a very important role. 4) For training data, existing methods do not consider different scores.

The number of values varies greatly, with scores of 4 and 5 taking a large proportion, and the training results.

It is unfair for low-score data, easy to cause overfitting, and the model is robust Difference. To address these issues, we propose a description based on review text and item descriptions.

The deep learning recommendation model described above.

The work of this paper can be summarized into the following three points:

1. Use the pre-trained BERT [18] model (bert_base_uncase) provided by Google to process the comment text instead of CNN, which overcomes the weakness that CNN can only extract local features, and can more accurately capture words in different contexts. Semantics, measuring the contribution of different comments to user characteristics, combined with forward. The Long Short-Term Memory (LSTM) model is used to learn the user’s interest migration over time, which improves the recommendation accuracy. Many models choose Bidirectional Recurrent Neural Network (RNN) to process the data, but for our model, the semantic information has been learned by BERT, and we only expect LSTM to learn the change of user interest over time. Since only existing reviews can influence future reviews, future reviews cannot influence existing reviews, and backward LSTM is effective in learning interest transfer. It does not work well and only increases the complexity of the model, so we do not use it.
2. Introducing item description documents together with reviews into the model helps us better describe item features and improve prediction accuracy, and when new items lack reviews, item description documents can well alleviate the cold start problem of items.
3. For the experimental data, we randomly sample the review data with the five scores of 1 to 5 at a ratio of 1:1:1:1:1 to ensure that the amount of data for each score is equal to reduce overfitting. Improve the robustness of the model.

Comparing experiments on four sets of public datasets, the results show that the prediction and scoring accuracy of our deep semantic mining-based recommendation model DSMR is higher than that of the current best review text-based models, such as DeepCoNN [10], NARRE [15], DER [17] et al.

## 2 Relate works

In recent years, the success of deep learning in natural language processing, computer vision and other fields has made the recommendation field begin to pay attention to this powerful tool, and scholars have begun to explore the use of deep learning methods to improve some insurmountable weaknesses of current recommendation systems, such as data sparseness, cold start, poor interpretability and other problems [19,20]. In particular, the emergence of CNN and RNN [21–26] has achieved great success in many natural language processing (NLP) tasks. So everyone began to try to use deep learning methods, such as DeepCoNN, D-Attn [12], etc., to mine user preferences and product characteristics in review texts, and then directly apply them to predictive scoring. DeepCoNN is composed of two parallel neural networks with CNN as the basic model, learning the implicit representation of users and items respectively, and connecting the two parts at the top of the network to learn interaction, which proves the effectiveness of review texts for alleviating the sparse problem.

The key to the attention mechanism [27] is to learn a weight to identify the degree of importance, which has been widely used in natural language processing since it was proposed, in machine translation [28,29], reading comprehension [30,31], speech Recognition [32] and other fields have achieved state-of-the-art results [33]. As a result, the attention mechanism has attracted the attention of the recommendation field and has been used in review-based recommendation algorithms [12,15,16,34]. NARRE [15] uses an attention mechanism to learn the usefulness of different reviews, better model users and items, predict item ratings and generate explanations. Different from the D-Attn word-level attention mechanism, NARRE adopts a comment-level attention mechanism. Inspired by Transformer [35], MPCN [16] does not use RNN and CNN, and completely relies on the At-tention mechanism, and proposes a new pointer-based learning scheme, which enables deep textual interaction between users and items. and achieved good results.

The development of NLP has greatly promoted the application of review texts in the field of recommendation. Pre-trained language models [14] have developed rapidly since they were proposed, resulting in many excellent methods, such as feature-based ELMo [36] and fine-tuning-based OpenAIGPT [37]. But these language models are unidirectional in nature, limiting the representational power of pretraining. Therefore, literature [18] proposed a two-way pre-training model BERT, which uses Transformer’s Encoder to read the entire text at one time, so that the model can learn based on both sides of the word, so as to more accurately grasp the expression of the word in the sentence meaning. Therefore, BERT has a natural bidirectionality and strong generalization ability, which provides a good foundation for downstream tasks.

## 3 DSMR model

### 3.1 Model frame

Each user buys many items and reviews many items, so we can use reviews as a representation of user preferences. But for the user, the description of the item is equally important, because only when the user is attracted by the description of the item will they choose to browse this item and see the reviews this item has received; in addition, for a new item, it has not been or is rarely purchased and evaluation, and the item description provides rich item attribute information, which helps to solve the problem of cold start of items. Many models only use the review text when modeling with text, and do not pay attention to the item description document. We think this will lose some important information, so we also input the item description into the model to get more accurate prediction results.

**Fig 1:**
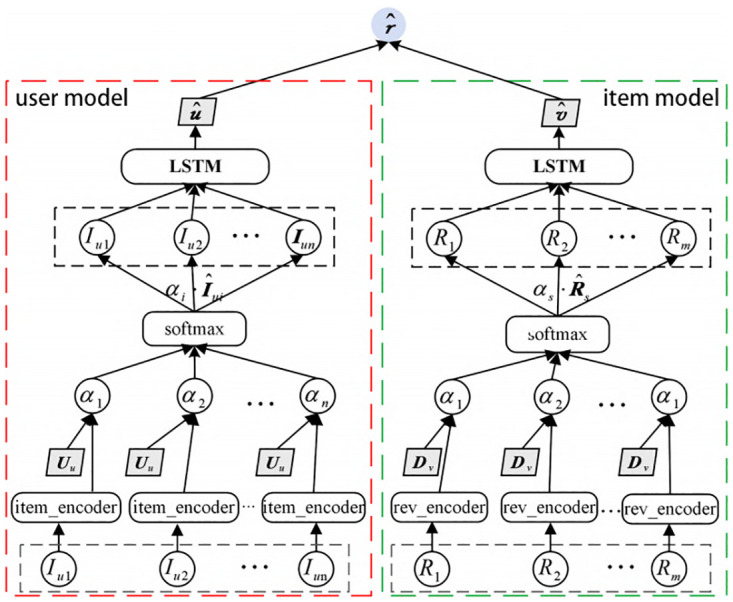
DSMR framework.

DSMR utilizes a BERT pretrained model to process textual data and distinguish the importance of different reviews, thereby helping us to more accurately predict a user’s rating for an item. The structure of the DSMR model is shown in Fig 1. The model is divided into two parallel parts, one is the user module and the other is the item module. In the user module, enter the description documents of all items reviewed for the user and all the comments received for each item; in the item module, enter all the comments received for this item and the description of this item. Finally, the results obtained by the two modules are dot-producted to obtain the user’s predicted score for this item. Since the structure of the user module and the item module is similar, this paper takes the user module as an example to introduce our model in detail.

### 3.2 Details

#### 3.2.1 Encode

**Fig 2:**
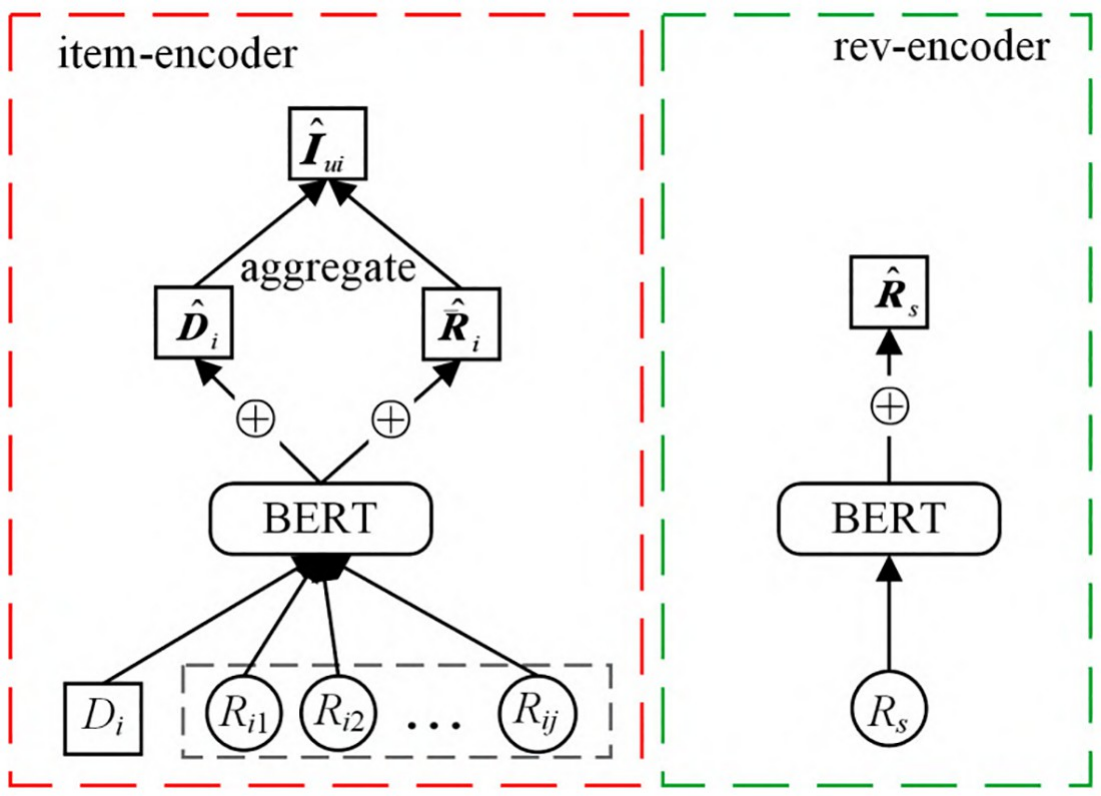
Encoder part.

For a user u, all items he has reviewed are represented by *I*_*ui*_(i = 1,2,…n). Pass *I*_*ui*_ into the item_encoder module. The specific structure of item_encoder is shown in the left frame of Fig 2, where ⊕ means addition. In the item_en-coder module, the description document Di of the item *I*_*ui*_ and all the comments *R*_*ij*_ (j = 1, 2,…, m) received by the item *I*_*ui*_ are passed into BERT. Our comparison model NARRE uses CNN to process comment text, and can only establish short-distance dependencies on the input sequence, while Self-attention in Transformer can process variable-length information sequences by dynamically generating weights of different connections, and can achieve parallelism to improve the training speed.

After the item description document *D*_*i*_ is pre-trained by BERT, the word vector representation of the item description is obtained, and the word vectors are added to obtain ${\hat{D}}_{i}$ Add to get ${\hat{R}}_{i}$, and combine ${\hat{D}}_{i}$ and ${\hat{R}}_{i}$ to get item embedding vector ${\hat{I}}_{ui}$, ${\hat{I}}_{ui}$ describes the characteristics of item i. The formula is as follows:

$${\hat{D}}_{i}=BERT(D_{i})$$ (1)

$${\hat{D}}_{i}=sum(BERT\left(R_{i1},R_{i2},{\ldots ,R}_{im}\right))$$ (2)

$${\hat{I}}_{ui}={\hat{D}}_{i}⊙{\hat{R}}_{i}$$ (3)

Among them, ⊙ means that the two vectors are concatenated.

For item v, all comments it receives are expressed as *R*_*s*_ (s = 1,2,…,m)

Representation, the review gets the review implicit representation ${\hat{R}}_{S}$ after passing through the BERT model, as shown in the rev_encoder part on the right side of Fig 2.

#### 3.2.2 LSTM

LSTM is mainly used to solve the long-term dependency problem in RNN (Recurrent Neural Network). LSTM is a special recurrent neural network so it also has a chain structure, but it has a different structure compared to the repeated modules of the recurrent neural network. It has four neural network layers, and each network layer interacts in a special way, Not a single simple neural network layer.

The state of each transmission unit is the core of determining the LSTM network. A unit state is equivalent to a conveyor belt, which runs through the entire structure. In this process, only some linear effects are used to ensure the invariance of information transmission. LSTM also has a good performance that can add and remove information transmitted to the unit state, manage the transmission of information through several structures and call it a threshold, the threshold is to selectively allow information to pass.

Models that use LSTM methods to explore user preferences over time perform better than models that do not focus on user preferences over time. LSTM preserves the error for backward pass along time and layers. LSTMs keep the error at a more constant level, allowing the recurrent network to learn over many time steps, opening up avenues for establishing long-range causal connections. LSTM can be used as complex nonlinear units to construct larger deep neural networks.

We use word embedding to represent user id as user embedding vector *U*_*u*_ (u = 1, 2,…, d), where d is the total number of users. Map *U*_*u*_ to the same space as the item embedding vector ${\hat{I}}_{ui}$ and perform dot product operation to obtain the correlation degree *α*_*i*_ between the features of user u and item i. The larger the value of *α*_*i*_, the higher the correlation degree, the more interested the user is in the item.


$$\alpha_{i}=U_{u}∙{\hat{I}}_{ui},i=1,2,\ldots ,n$$ (4)

Normalize *α*_*i*_ (i = 1, 2,…,n) by softmax, and multiply the normalized *α*_*i*_ by ${\hat{I}}_{ui}$ to get the contribution degree of each item to user characteristics.

Finally, ${\hat{I}}_{ui}$ is sent to LSTM to learn the user’s interest migration over time, and the output vector $\hat{u}$ of the user model is obtained.


$$\hat{u}=(LSTM\left(softmax\left(\alpha_{i}\right)∙{\hat{I}}_{ui}\right),i=1,2,\ldots ,n$$ (5)

Similarly, we denote the description document of the item *v* as *D*_*v*_, and map *D*_*v*_ and the item comment embedding vector ${\hat{R}}_{s}$ to the same space for operation, and the output vector $\hat{v}$ of the item model can be obtained.

#### 3.2.3 Rating prediction

Do the dot product between the output vector $\hat{u}$ of the user model and the output vector $\hat{v}$ of the item model to get the final prediction score $\hat{r}$.


$$\hat{r}=\hat{u}∙\hat{v}$$ (6)

#### 3.2.4 Model training

The goal of the DSMR model is actually to improve the accuracy of score prediction, which is equivalent to a regression problem. For regression problems, the most commonly used objective function is the squared loss function. In the training set sample *M*, the predicted score of user *u* for item *i* is ${\hat{R}}_{ui}$, and the real score is *R*_*ui*_, then the objective function can be expressed as:

$$L=\sum_{u,i\in M}({{\hat{R}}_{ui}-R_{ui})}^{2}$$ (7)

Our task is to minimize the objective function. We choose the Adam [38] optimization algorithm to optimize the objective function, because Adam uses momentum and adaptive learning rate to speed up the convergence, is suitable for problems with large amounts of data and requires very little memory.

## 4 Modeling

### 4.1 Data set

In the selection of datasets, we refer to the datasets used by the most advanced models in the literature [15,16], and select four commonly used datasets from Amazon’s public datasets as our data: Movies_and_TV, Toys_and_Games, Kindle_Store and Videos_Games datasets. The basic statistical information is listed in Table 1.

**Table 1:** Statistics of dataset.

Dataset	Users	Items	Reviews
Movies_and_TV	123960	50052	1679533
Toys_and_Games	19412	11924	167957
Kindle_Store	68223	61935	982619
Videos_Games	24303	10672	231780

In the process of processing the dataset, we consider that although there are 5 points of 1–5 points, 5 points and 4 points still account for the majority of the scores, which is not considered by almost all the proposed models. We think this is unfair for 1- or 2-point data and will overfit the training results. We randomly extract the data of the five scores from 1 to 5 according to 1:1:1:1:1, so that the data of each score in the data set is equal, the results are more objective, and the model is more robust.

### 4.2 Model comparison

To verify the effectiveness of the models, we select 2 early classic models that only utilize rating matrices and 3 recently proposed advanced models that utilize review text as comparison models.

MF [39]: Matrix factorization is a very popular recommendation method based on collaborative filtering. It only uses the rating matrix as input, uses the inner product of the user and item low-rank matrices to represent the rating, and uses the alternating least squares (ALS) technique to minimize its objective function.

PMF [40]: Probabilistic matrix factorization is a traditional matrix factorization method, which only uses rating data for collaborative filtering, and introduces Gaussian distribution to model latent factors of users and items.

DeepCoNN: Taking CNN as the basic model, it consists of two parallel neural networks, one of which uses the user review set to learn user behavior, and the other parallel network uses the item review set to learn item attributes. An additional shared layer on top of the two neural networks connects the two parallel networks, enabling the learned user and item latent factors to interactively predict ratings. This model proves that the sparsity problem can be effectively alleviated by utilizing the review text.

NARRE: On the basis of DeepCoNN, the attention mechanism is used to judge the contribution degree of a review, and the accuracy and interpretability of the model are improved by selecting more useful reviews for modeling.

DER: Similar to the first two models, DER also uses CNN to extract item attributes. In addition, DER believes that the traditional GRU does not consider that the user’s interest will change after a large time interval, so it proposes to improve the GRU by adding a time gate, so as to more accurately predict the user’s current preferences.

In addition, we also set up a comparative model review-DSMR, which is based on the DSMR proposed in this paper but only uses the review text and does not add the item description document to the recommendation model, in order to verify the promotion effect of the item description document on the recommendation effect.

### 4.3 Evaluation indicators

We use the root mean square error (RMSE), which is widely used in algorithm performance evaluation, as the evaluation index. The formula is as follows:

$$RMSE=\sqrt{\frac{1}{N}\sum_{n=1}^{N}({{\hat{R}}_{u,i}-R_{u,i})}^{2}}$$ (8)

Among them, *N* is the number of samples in the test set, ${\hat{R}}_{u,i}$ is the predicted rating of item *i* by user *u*, and *R*_*u*,*i*_ is the actual rating of item *i* by user *u*. The smaller the value of RMSE, the better the performance of the model.

### 4.4 Parameter settings

After the data is randomly sorted, 70% is used as the training set, 20% is used as the validation set, and 10% is used as the test set. The BERT pre-trained model we use is bert_base_uncase trained by Google, and the review-DSMR and DSMR models have an initial learning rate of 0.01, which is then dynamically adjusted using the NoamOpt optimizer. The loss rate is set to [0.05, 01, 0.3, 0.5], the batch size is set to [3, 5, 8, 16, 32], and the number of latent factors is set to [32, 64, 128, 256].

For MF and PMF, we use grid search to find the best value of latent factor from [25, 50, 100, 150, 200] according to the setting strategies of [33] and [34], respectively, from [0.001, 0.01, 0.1, 1.0] to find the optimal value of the regularization parameter. For DeepCoNN and NARRE, we reproduced according to the settings of the literature [10, 15] respectively, the learning rate was [0.005, 0.01, 0.02, 0.05], and the batch size was [50, 100, 150], the loss rate is [0.1, 0.3, 0.5, 0.7, 0.9], and the number of latent factors is [8, 16, 32, 64] [15]; for CNN text processors, The number of neurons in the convolutional layer is 100 and the window size is 3. For the comparative model DER, the learning rate is set to [0.001, 0.01, 0.1, 1], the batch size is [50, 100, 150], and the user/item embedding size is [8, 16, 32], [64, 128] to adjust.

In order to verify that the 1:1:1:1:1 equivalent control of the training data can improve the accuracy of the algorithm, we conducted experiments on all models without the equivalent control and with the equivalent control. Experimental results.

### 4.5 Results and analysis

After many experiments, the DSMR model works best when the loss rate is 0.1, the batch size is 5, and the number of latent factors is 128. The experimental results of each model are listed in Tables 2 and 3.

**Table 2:** Performance comparison without data equal control (RMSE).

	Movies_and_TV	Toys_and_Games	Kindle_Store	Videos_Games
MF	1.522	1.379	1.286	1.503
PMF	1.276	1.158	1.102	1.311
DeepCoNN	1.193	1.044	1.025	1.231
NARRE	1.147	1.008	0.976	1.192
DER	1.106	0.983	0.942	1.145
review-DSMR	1.098	0.977	0.913	1.115
DSMR	1.073	0.935	0.884	1.097

**Table 3:** Performance comparison with data equalization control (RMSE).

	Movies_and_TV	Toys_and_Games	Kindle_Store	Videos_Games
MF	1.357	1.239	1.208	1.415
PMF	1.122	1.026	0.974	1.206
DeepCoNN	1.107	0.993	0.955	1.154
NARRE	1.075	0.974	0.937	1.141
DER	1.049	0.954	0.902	1.109
review-DSMR	1.035	0.921	0.874	1.083
DSMR	1.017	0.897	0.839	1.058

**Fig 3:**
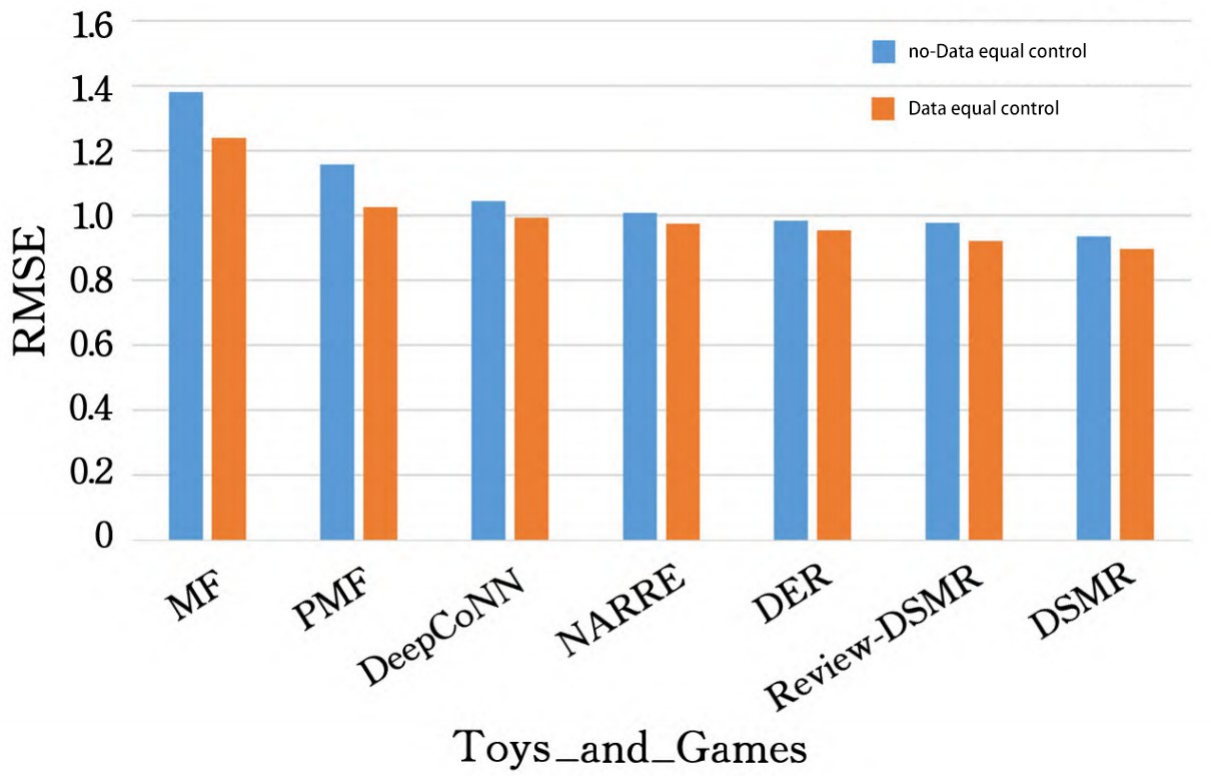
Effect comparison with/without data equal control.

**Fig 4:**
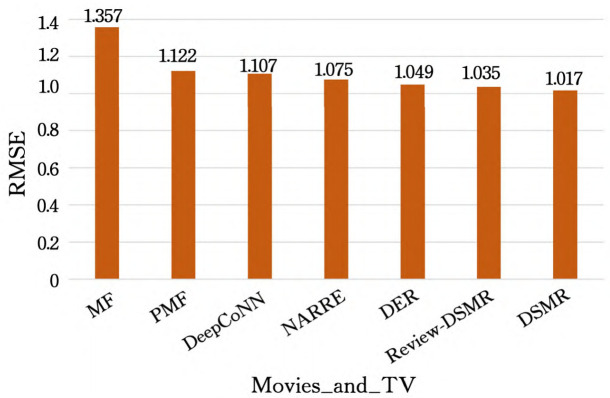
Performance comparison with data equal control.

In order to make the experimental results more intuitive, we made Tables 2 and 3 into Fig 3 (taking the Toys_and_Games dataset as an example) to show the performance difference with and without data equal control, and displayed Table 3 in the form of a histogram come out (see Fig 4), to facilitate the analysis of various aspects later (take Movies_and_TV as an example).

As can be seen from Fig 3, after the training data of the five scores of 1 to 5 points of all models are extracted in equal amounts of 1:1:1:1:1, the RMSE of all models is compared with no data equalization control. The time is reduced, which proves that equal control of training data can help to improve the recommendation effect. Because there are few comments with low scores for the data that are not processed, and the data with 4 and 5 scores are the majority, the model is easy to overfit, so after equal processing, the model is more robust.

As can be seen from Table 3, when all models are controlled with equal amount of data, the DSMR model is still better than the previous state-of-the-art models, and the RMSE is better than the MF, PMF, DeepCoNN, NARRE and DER models in 4. The average reductions on each dataset are 26.98%, 11.95%, 9.46%, 7.66% and 5.1%, respectively.

The following is an analysis of the influence of the information based on each model and the extracted features on the experimental results. First of all, the model using the review text is better than the traditional model using only the rating data. As can be seen from Fig 4, the RMSE of DeepCoNN, NARRE, DER and DSMR are all lower than MF and PMF, which proves that the review data is not effective. It is beneficial for the model to learn more accurate user characteristics and item attributes, and it does promote the improvement of recommendation accuracy.

Secondly, for the models that also consider the comment text, the model with the attention mechanism is better than the model without the attention mechanism. For example, the RMSE of NARRE, DER and DSMR is lower than that of DeepCoNN, because the attention mechanism can learn The contribution of each review to user characteristics and item attributes, so using different reviews with different weights is better than using all reviews indiscriminately.

Furthermore, the model using the BERT pre-training method is better than the model using static word vectors. For example, the RMSE of DSMR is lower than that of DeepCoNN and NARRE, because BERT can learn different meanings of words in different contexts, while static word vectors It cannot, so this makes the effect of feature extraction between the two very different. In addition to DeepCoNN, both NARRE and DER use CNN to extract features, while CNN can only learn local features, and the information loss for long sequences is relatively large. Furthermore, the DSMR model utilizes the review text and also introduces the item description document, which is ignored by other models. The item description document not only enriches the item attribute information, but also alleviates the cold start problem of the item.

Finally, models that use the LSTM method to explore user preferences over time perform better than models that do not focus on user preferences over time, such as DER (using GRU) and DSMR with lower RMSE than DeepCoNN and NARRE.

**Fig 5:**
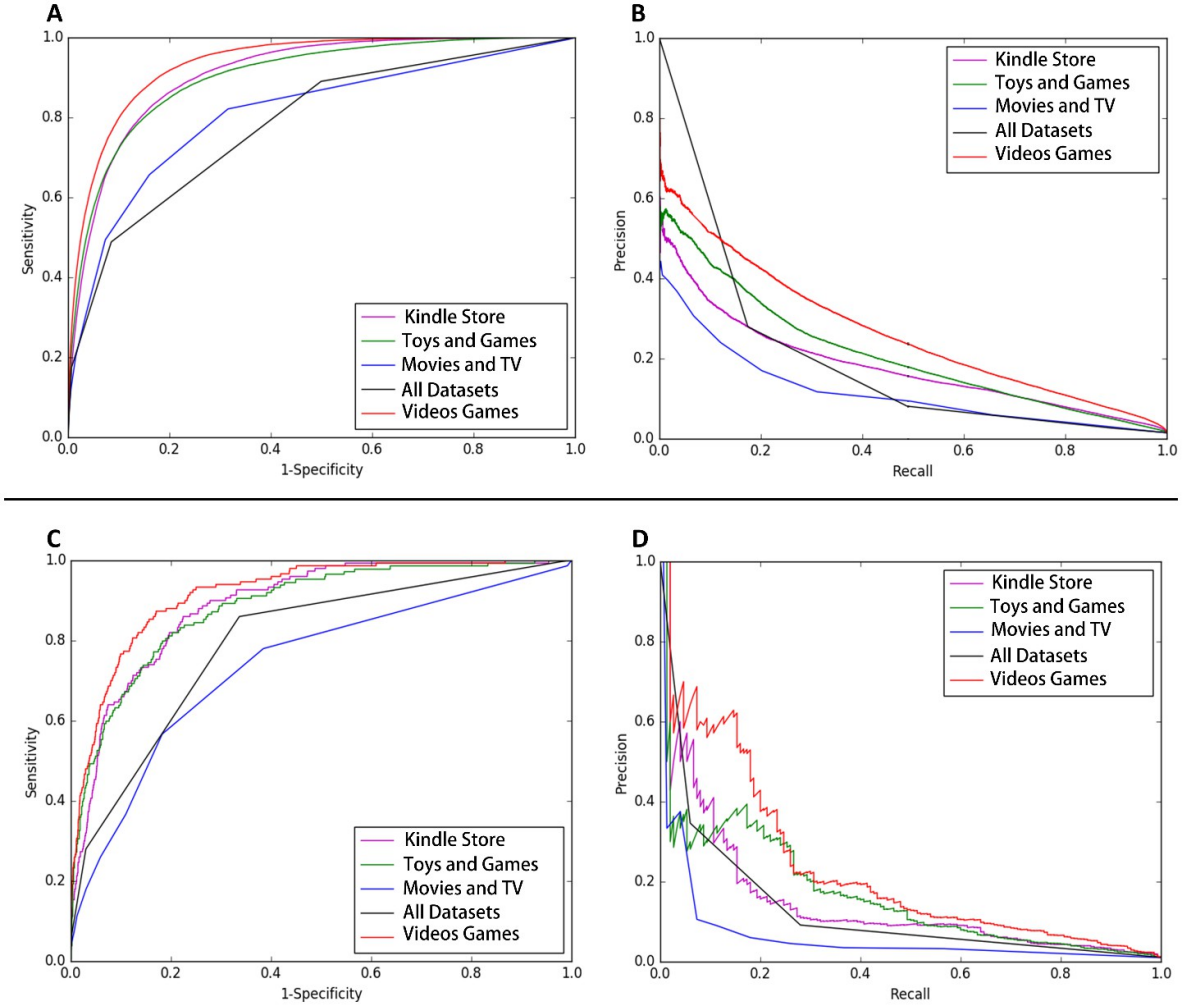
Effect comparison with/without item description.

In Fig 5, Figure A is the ROC curve of review-DSMR (AUC: Kindle Store: 0.910, Toys and Games: 0.903, Movies and TV: 0.810, All Datasets: 0.785, Videos Games: 0.935), Figure B is the precision of review-DSMR -recall curve, Figure C is the ROC curve of DSMR (AUC: Kindle Store: 0.822, Toys and Games: 0.818, Movies and TV: 0.726, All Datasets: 0.797, Videos Games: 0.894), Figure D is the precision-recall curve of DSMR.

**Fig 6:**
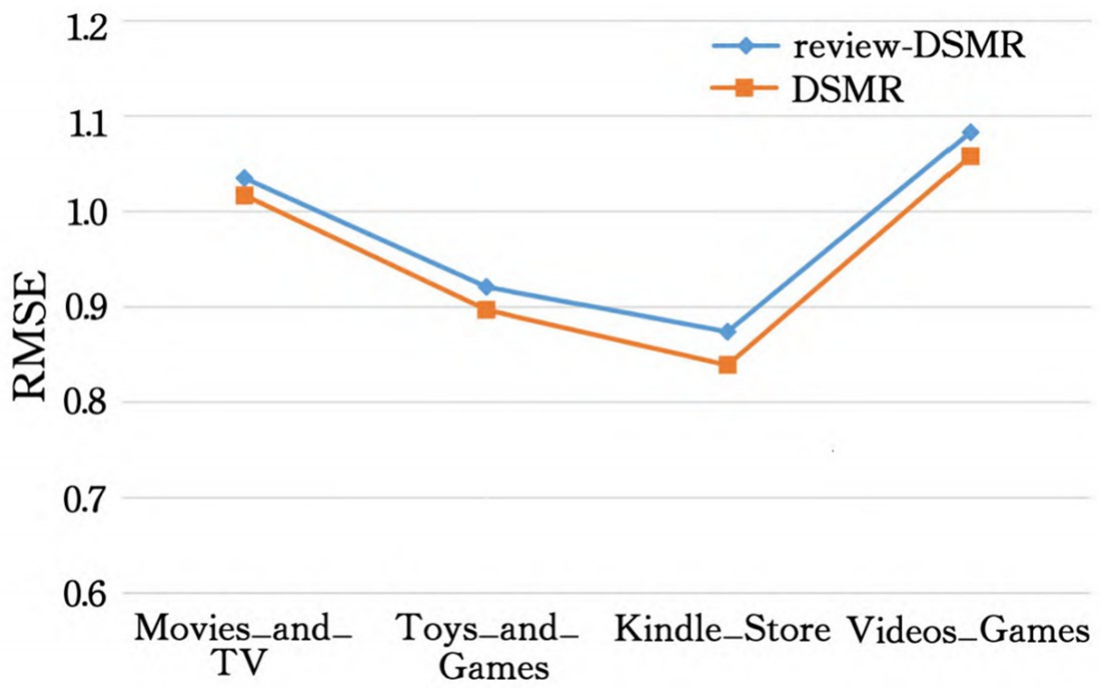
Comparison of roc and precision-recall between review-DSMR and DSMR.

From the comparison of the experimental results of review-DSMR and DSMR in Fig 6, it can be seen that the RMSE of the DSMR model with the addition of the item description document is significantly lower than that of the review-DSMR model that only uses the comment text, which proves that the item description document is indeed conducive to enriching item attributes, so as to improve the recommendation accuracy.

## 5 Conclusion

This paper proposes a deep semantic mining recommendation model that can more accurately predict ratings. It uses the BERT pre-training model to learn the more accurate semantics of words in contextual information and evaluate the importance of reviews. At the same time, item description documents are introduced to alleviate the cold start problem of items, and also use LSTM to learn the internal relationship between reviews, explore the changes of user preferences over time, and use equal extraction of each score in the experimental data processing to improve the robustness of the model. Experimental results show that the DSMR model is 5.1% higher than the current state-of-the-art review text-based recommendation model in terms of predictive rating accuracy.

## Supporting information

S1 File (ZIP)

## References

[1] KIM D, PARK C, OH J, et al. Convolutional matrix factorization for document context-aware recommendation[C]//Proceedings of the 10th ACM Conference on Recommender Systems. ACM, 2016:233–240.

[2] WANG C, BLEI D M. Collaborative Topic Modeling for Recommending Scientific Articles[C//Proceedings of the 17th ACM SIGKDD International Conference on Knowle date Discovery and Data Mining.ACM2011:21–24.

[3] MCAULEY J, LESKOVEC J. Hidden factors and hidden topics: understanding rating dimensions with review textC1//Proceedings of the ACM Conference on Recommender Systems. ACM 2013:165–172.

[4] BAO YFANG H, ZHANG J. Topicmf: Simultaneously exploiting ratings and reviews for recommendation[C//Proceedings of the Twenty-Eighth AAAl Conference on Artificial Intelligence. AAAl Press. 2014:2–8.

[5] TAN YZHANG MLIU Yet al. Rating-boosted latent topics: Understanding users and items with ratings and reviews[C1//Proceedings of the Twenty-Fifth international Joi nt Conference on ArtificialIntelligence 2016:2640–2646.

[6] LING GLYU M RKING 1.Ratings meet reviews, a combined approach to recommend[C//Proceedings of the ACM Conference on Recommender Systems(RecSys). A CM. 2014:105–112.

[7] CATHERINE R, COHEN W. Transnets: Learning to transform for recommendation[C]/Proceedings of the 11th ACM Conference on Recommender Systems. ACM, 2017:288–296.

[8] BLEI D M, NG A ORDAN I. Latent dirichlet allocation[J].Journal of Machine Learning Research, 2003,3(4/5):993–1022.

[9] LEE D D, SEUNG H S. Algorithms for Non-negative Matrix Factorization[C]//International Conference on Neural Information Processing Systems. MIT Press,2000:556–562.

[10] ZHENG LNOROOZIV. YU P S. Joint deep modeling of users and items using reviews for recommendationC1//Proceedings of the Tenth ACM International Conference on Web Search and Data Mining.ACM2017:425–434.

[11] KIM DPARK C. OH J, et al. Convolutional Matrix Factorization for Document Context-Aware Recommendation [C]//ACM ConferenceACM. 2016:233–240.

[12] SEO S. HUANG J. YANG H. et al Interpretable Convolutional Neural Networks with Dual Local and Global Attention for Review Rating Prediction[C]//The Eleventh AC M Conference. ACM. 2017:297–305.

[13] WU L QUAN C, L C, et al. A context-aware user-item representation learning for item recommendation[J]. ACM Transactions on Information Systems (TOIS), 2019,37 (2):1–29.

[14] DAI A M, LE Q V. Semi-supervised Sequence Learning [J.MIT Press, 2015.

[15] CHEN C. ZHANG M. LIU Yet al Neural attentional rating regression with review-level explanations[C]//Proceedings of the 2018 World Wide Web Conference.2018:1583–1592

[16] TAY YLUU A THUIS C. Multi-pointer co-attention net-works for recommendation[C]/Proceedings of the 24th ACM SIGKDD International Conference on Knowledge Discovery & Data Mining.2018:2309–2318.

[17] CHEN XZHANG YQIN Z. Dynamic Explainable Recommendation Based on Neural Attentive ModelsJ1. Proceedings of the AAAl Conference on Artificial Intelligence 2019,33:53–60.

[18] DEVLIN J, CHANG M WLEE K, et al. BERT: Pre-training of Deep Bidirectional Transformers for Language Understanding[U]. arXiv:1810.04805, 2018.

[19] CAO S,YANG N,LIU Z. Online news recommender based on stacked auto-encoder[C]//ACIS 16th International Conference on Computer and Information Science (IClS). IEEE.2017:721–726.

[20] WANG H. WANG N. YEUNG D Y Collaborative Deep Learning for Recommender Systems[C]//KDD 2015. ACM. 20151235–1244.

[21] Kim DW, Jang HY, Ko Y, et al. Inconsistency in the use of the term “validation” in studies reporting the performance of deep learning algorithms in providing diagnosis from medical imaging[J]. Plos one, 2020, 15(9): e0238908. doi: 10.1371/journal.pone.0238908 32915901 PMC7485764

[22] Prakash AJ. Capsule Network for the Identification of Individuals Using Quantized ECG Signal Images. IEEE Sensors Letters. 2022 Aug 1;6(8):1–4.

[23] Hammad M., Chelloug S.A., Alkanhel R., Prakash A.J., Muthanna A., Elgendy I.A. and Pławiak P., 2022. Automated Detection of Myocardial Infarction and Heart Conduction Disorders Based on Feature Selection and a Deep Learning Model. Sensors, 22(17), p.6503. doi: 10.3390/s22176503 36080960 PMC9460171

[24] Allam, J.P., Samantray, S., Behara, C., Kurkute, K.K. and Sinha, V.K., 2022. Customized deep learning algorithm for drowsiness detection using single-channel EEG signal. In Artificial Intelligence-Based Brain-Computer Interface (pp. 189–201). Academic Press.

[25] Sahoo J.P., Prakash A.J., Pławiak P. and Samantray S., 2022. Real-Time Hand Gesture Recognition Using Fine-Tuned Convolutional Neural Network. Sensors, 22(3), p.706. doi: 10.3390/s22030706 35161453 PMC8840381

[26] Locharla, G.R., Pogiri, R. and Allam, J.P., 2022. EEG-based deep learning neural net for apnea detection. In Artificial Intelligence-Based Brain-Computer Interface (pp. 203–215). Academic Press.

[27] BAHDANAU D,CHO K,BENGIO Y. Neural Machine Translation by Jointly Learning to Align and Translate[J]. arXiv:1409.0473,2014.

[28] GEHRING J,AULI M,GRANGIER D, et al. Convolutional sequence to sequence learning[J]. arXiv:1705.03122,2017.

[29] BAHDANAU D.CHO K.BENGIO Y Neural Machine Translation by Jointly Learning to Align and Translate[J]. Computer Ence, 2014.

[30] HERMANN KMKOCISKYTGREFENSTETTE E, et al. Teaching machines to read and comprehend[C]//Advances in Neural Information Processing Systems. MIT Press. 2015:1693–1701.

[31] SEO M, KEMBHAVI A,F ARHADI A, et al. Bidirectional attention flow for machine comprehension[J].arXiv:1611.01603, 2018.

[32] AMODEID, ANANTHANARAYANAN SANUBHAIR et al. Deep Speech2:End-to-End Speech Recognition in English and Mandarin[C]/ICML. 2015.

[33] LU Y, DONG RSMYTH B. Coevolutionary recommendation model: Mutual learning between ratings and reviews[C]//Proceedings of the 2018 World Wide Web Conference. 2018:773–782.

[34] CHEN J. ZHANGHHE X.et al. Attentive Collaborative Filtering: Multimedia Recommendation with Item-and Component-Level Attention[C//International ACM Sigir Conference ACM 2017:335–344.

[35] VASWANI A, SHAZEER N, PARMARN, et al. AttentioniskCM0IAll You Need[J]. arXiv:1706.03762,2017.

[36] PETERS M, NEUMANN M, IYYER M et al. Deep Contextualized Word Representations[C]//Proceedings of the 2018 Conference of the North American Chapter of the Association for Computational Linguistics: Human Language Technologies, Volume 1 (Long Papers). 2018.

[37] RADFORD A, NARASIMHAN KSALIMANS T., et al. lmproving language understanding with unsupervised learning [R]. Technical report, Open Al, 2018.

[38] KINGMA D, BA J. Adam: A Method for Stochastic Optimization[J]. arXiv:1412.698,2014.

[39] KOREN Y BELL R VOLINSKY C. Matrix Factorization Techniques for Recommender Systems[U]. Computer, 2009,42(8):30–37.

[40] SALAKHUTDINOV R, MNIH A. Probabilistic matrix factorization[C]//Proceedings of the 20th International Conference on Neural Information Processing Systems. 2007:1257–1264.

